# The current situation for gastric cancer in Chile

**DOI:** 10.3332/ecancer.2016.707

**Published:** 2016-12-21

**Authors:** Christian Caglevic, Shirley Silva, Mauricio Mahave, Christian Rolfo, Jorge Gallardo

**Affiliations:** 1 Cancer Drug Research Unit, Fundación Arturo López Pérez, Santiago, Chile; 2 Radiation Oncology, University of Valparaíso, Valparaíso, Chile; 3 Medical Oncology Service, Fundación Arturo López Pérez, Santiago, Chile; 4 Early Drug Development Unit – Phase I, University Hospital of Antwerp, Antwerp, Belgium; 5 Fundación Chilena Desarrollo Oncología, SLAGO (Latin American Symposium on Oncological Gastroenterology), Santiago, Chile.; 6 Instituto Oncológico Fundación Arturo López Pérez, Rancagua 878, Providencia Santiago, Chile

**Keywords:** gastric cancer, Chile, epidemiology, investigation, gastric cancer treatment in Chile

## Abstract

Gastric cancer is a neoplasm with a high incidence and mortality rate in Chile where more than 3000 people die every year from this type of cancer. This study shows the clinical and epidemiological considerations of this disease, information about translational research on this pathology in Chile, the contribution of Chilean doctors to the development of gastric cancer management awareness and the general situation of gastric cancer in Chile.

## Introduction

The incidence of gastric cancer worldwide has experienced an important decrease in recent years [1] going from being the most common neoplasm in the middle of the 1970s to being currently in 5^th^ place; however, it is still one of the main causes of cancer deaths, representing the third cause of death by malignant tumours in both sexes at present [2].

In Chile, gastric cancer is the main cause of cancer deaths. Its incidence and mortality estimated by the IARC is 15.6 and 13.8 per 100,000 inhabitants, respectively [2]. According to national statistics, in 2012, there were 3,354 deaths by gastric cancer with a mortality rate of 19.27 per 100,000 inhabitants for both sexes (25.3 and 13.3 per 100,000 inhabitants for men and women, respectively) [3]. These latest statistics are very similar to the highest worldwide mortality rates, which are found in East Asia where a mortality rate of 24 per every 100,000 men and 9.8 per every 100,000 women is estimated [2]. Given the importance of stomach cancer in Chile, it is relevant to emphasise some epidemiological phenomena that have occurred worldwide in recent years and associate them with what has been happening in our country, in addition to emphasising the advances that stand out in the investigation of this pathology and discussing some general aspects of the treatment in our field.

## Epidemiology and pathological considerations

The majority of malignant stomach tumours correspond to the histological type of adenocarcinoma (90% approximately), with a lower percentage of MALT lymphomas, leiomyosarcomas and other rarer tumours [1]. The adenocarcinomas have been divided classically into two histological subtypes: (a) diffuse and (b) intestinal types [4], each of which has differences in their presentations depending on the anatomic subsite, age when diagnosed, sex, race, demographical distribution and socio-economic situation. The intestinal type, typically with distal (not cardia) location, prevails in developing countries, in black people, in lower socio-economic groups, and it has also been associated with chronic infection by *Helicobacter pylori (H. pylori)*. This subtype has experienced an important decrease in recent years, which explains a decrease in the worldwide incidence [5]. On the other hand, the diffuse type, with a proximal location (cardia), prevails in developed countries, in men, in white people, in higher socio-economic levels, and it has been associated with gastroesophageal reflux disease and obesity. There is currently an increase in the incidence of this subtype worldwide [6].

In Chile, an increase has been reported of the cardia location of gastric cancer but with reports of incidence rates that range between 12% and 45% of the total [7, 8]. Regional differences have also been reported, emphasising the rise in the diffuse type of up to 55% in the southern part of the country [9].

Cardial tumours have worse survival rates and higher operative mortality [6], as well as being associated with polymorphisms of certain genes in high prevalence populations, such as variants of the XRCC1 gene (genotypes 26304 CC and 28152 AA/GA) found in the Chinese population, which are associated with a greater risk of gastric cancer in this specific location [10]. Given the increase in cardial cancer in our population, the investigation in our country could provide relevant details regarding the etiopathogenesis of the disease, as well as predictive and prognostic factors. It is interesting that the prospective cohort study MAUCO [11], which is currently ongoing, includes the population of the Molina commune (city in the southern-central area of Chile that has a high incidence of gastric cancer). This study will investigate exposure factors related to chronic diseases and will make way for the creation of a biobank, laying the foundations for possible population and genetic studies in patients with stomach cancer.

## Environmental and racial factors associated with gastric cancer in Chile

Research into gastric adenocarcinoma etiopathogenesis has enabled us to understand the development of gastric cancer as a step-by-step process in the intestinal subtype, which progresses through stages from a chronic superficial gastritis, chronic atrophic gastritis, intestinal metaplasia, dysplasia and finally cancer formation [12]. This process has been associated with multiple factors, the most significant being infection by *H. pylori* [13, 14] and a high salt consumption [15]. In Chile, there is a high prevalence of *H. pylori* in adults and children (prevalence rates of 73% and 18.1%, respectively, have been reported) [16], and a high salt consumption of around 9 g a day [17], which in addition to encouraging infection by *H. pylori*, increases the risk of developing gastric cancer [15]. Another important precedent to consider is that 80% of the population of Chile have a sedentary lifestyle, 25% suffer obesity [18] and this situation has been indirectly associated with the development of proximal gastric cancer [19].

The Epstein-Barr virus (EBV) has received special interest in recent years. The presence of this virus is found in approximately 8.7% of all gastric cancers. It has been possible to research the presence of monoclonal EBV in neoplastic cells, and it has been suggested that there is an epigenetic alteration mechanism involved in carcinogenesis [20]. Within the relevant characteristics of gastric carcinoma associated with EBV, it is emphasised that the prevailing location is in the cardia [21].

As has already been mentioned, cancer located in the cardia has increased its incidence in recent years, which has awoken the interest of national researchers. It has been possible to identify the presence of EBV in 16.8% in a Chilean cohort of gastric cancer patients, which represents one of the highest rates worldwide, with a prevailing cardial location (26.9%) and without significant differences according to histological subtypes [22]. Strangely enough, despite this being possibly associated with the etiopathogenesis of the disease, in national publications, a tendency has been observed of greater survival rates in patients with VEB (+) when compared with patients VEB (-), being otherwise in accordance with international reports [7].

There has been an increase in incidence and mortality in some indigenous populations worldwide, such as the Inuit, Maori and Mapuches [23]. The Mapuche people are the largest indigenous group in Chile, and it is estimated that they represent nearly 10% of the country’s total population and approximately 80% of the indigenous population [24]. Despite a notoriously greater incidence of gastric cancer in the Mapuche population according to international studies, in national studies, this risk factor has only been proved among women with Mapuche ancestry [25]. Nevertheless, the fact that the second highest mortality rate in our country is found in the region of Araucanía, ancestral territory of the Mapuche people, cannot be ignored [3].

## Research into gastric cancer and translational medicine

Science has enabled us to understand that the etiopathogenesis of cancer is a complex, dynamic process that is still not fully understood. Current technology, progress in genomic sequences, and new molecular techniques have made the study of the complex quantity of genes and multiple pathways involved possible, making distinctions between genetic and epigenetic alterations involved in the tumorigenesis.

We know that in the majority of cases of gastric cancer, a hereditary precedent cannot be found, and in the majority of family cases, which represent no more than 15% of the total number of patients with this pathology, it has not been possible to establish a germline mutation [26]. Notwithstanding the above, in some family syndromes, it has been possible to identify mutations such as in the Hereditary Gastric Cancer Syndrome and alterations in the CDH1 gene [27]. Also in gastric intestinal cancer and in patients with non hereditary polyposis colon cancer alterations in the DNA reparation genes ADN MSH2 and MLH1 have been identified. Nevertheless, these alterations are not frequent, which indicates that the etiopathogenesis of gastric cancer is a much more complex process than a point mutation. Acquired genetic factors seem to have a fundamental role in the development of gastric cancer, including alterations that generate chromosomal instability, such as aneuploidy, translocation, amplification, deletion or loss of heterocygosity and gene fusion. It is also important to know the heritable changes that determine the expression of genes without altering the primary sequence of the DNA, which has been termed epigenetic [28].

One of the most important epigenetic alterations described is microsatellite instability. Microsatellites are sequences of usually noncoding DNA that are highly repetitive and numerous. When the process of replication is produced, there can be sequence slippage, which generates mutations that are normally corrected by the DNA repair mechanism, formed by encoded protein from several genes, with the hMLH1 being especially important. If these genes are inactivated for different causes, for example due to hypermethylation in its promoter region, mutations in the sequences are accumulated, creating the phenomenon of microsatellite instability. This way the microsatellite instability reflects alterations in the machinery of cell repair that can produce determining alterations in the development of the neoplasm such as the inactivation of suppressor genes; hence, the identification of this could be used as a potential investigational test for this disease [29].

Other epigenetic mechanisms associated with the methylation of DNA have been studied; in particular, the study of the Reprimo gene has opened an interesting line of investigation. The Reprimo gene located in the chromosome 2q23.3 codes a highly glycosylated p53-mediated protein, being able to cause abnormal cells to stop at G2 phase. It is believed that it regulates the activity of the complex cdc2/cyclin B1 and prevents the uncontrolled proliferation of the cell [30], its inactivation is produced by DNA methylation and is found present in multiple tumours in the early stages of the disease. In gastric cancer studies, this gene has been found in methylated form in up to 95% of tumours studied [31] and, in more recent series, these high percentages of methylation have been confirmed, accompanying also the methylation of the hMLH1 gene, and even being found present in earlier stages of the disease both in the plasma and the tissues [30]. For these reasons, the Reprimo gene has become of interest to several national groups in order to study it as a marker in screening gastric cancer, and as a prognostic and predictive factor. Such is the case of the DEMRAC (NCT01774266) study. Its goal is to evaluate the detection of Reprimo in plasma, determining its specificities and sensitivity in the detection of gastric cancer in a population with high prevalence, in addition to characterising the clinical, molecular and pathological factors of the cases of cancer both identified and not identified by Reprimo, and also other factors of clinical relevance like the detection of *H. pylori* and gastric atrophy [32]. Several research centres have joined efforts in order to evaluate the detection of Reprimo methylated in plasma as a marker of response to preoperative chemotherapy in locally advanced gastric cancer [33].

## The treatment of gastric cancer in our country (Chile)

Despite the fact that advances in the understanding of gastric cancer in our country promise to be the first step towards improved quality in the treatment of these patients, there are still problems left to resolve. Even though the genetic differences in etiopathogenesis and the evolutionary theory depending on the histological subtype source are known, so far, there are no substantial differences in the management of these different groups of patients. Notwithstanding the above, for more than a decade, important scientific studies have been carried out in Chile that have contributed to global knowledge and have served to improve the management of patients with gastric cancer. It is important to emphasise the contributions of Csendes in the surgical area of patients with gastric cancer [34] and of Baeza as a pioneer in adjuvant radiochemotherapy of this same disease [35].

In recent years, more efforts have been made in the interest of improving access and treatment opportunities for different health problems, and this is why in 2004 Law Nr 19.966 was passed. This establishes a health-care guarantee scheme GES-AUGE [36], and since 2006, gastric cancer has been included, with the aim of guaranteeing its diagnosis and appropriate treatment [37]. However, the expiration of established deadlines for care and treatment is a common problem, with different fulfilment percentages in different regions. After analysing all the guarantees contemplated in the AUGE programme, what stands out is a delay in the meeting of deadlines of up to 60.3% in 2015 in the metropolitan region (the most densely populated region of Chile) [38]. This situation added to the estimates of unresectable or metastatic gastric cancer at the time of diagnosis, which varies according to different authors between 37% and 58% [39], implies that we are often too late to offer a potentially curative treatment.

A low educational level has been established as one of the determining social factors associated with gastric cancer in our country [25, 40]. It becomes evident that there is a connection between this factor combined with low income and the divide in our health system. After the 1980s, a reform of the Chilean health system was implemented, which made way for two principal systems: (a) a public one and (b) a private one. In the public sector, a national health fund ‘Fondo Nacional de Salud (FONASA)’ was created, which raises, administers and distributes the state resources to finance the network of the Sistema Nacional de Servicios de Salud (national health system). In its private counterpart, the Instituciones de Salud Previsional (ISAPRES) (health insurance institutions) were created. These are institutions in charge of offering or acting as agents to finance health-care services [41]. FONASA is used by 78.3% of the Chilean population and includes the sectors of lower income and homeless people, whereas ISAPRES attend to 14.2% of the population and its contributing members are among those with higher socio-economic levels [42]. This structure tends to create inequalities with regard to access and the quality of service; so for this reason, there are certain differences regarding the treatment of gastric cancer in the different systems. To give an example, surgery is the fundamental pillar and the principal modality of treatment in resectable diseases [43]. It is known that, in specialised centres with a large number of oncological surgeries per year, there are greater possibilities of achieving better oncological results [44, 45]. In cancers that are locally advanced, the perioperative chemotherapy of the MAGIC scheme or something similar [46] is the prevailing choice in private centres, but it is not always available in public centres because of its higher cost. It is because of this that surgery followed by radiochemotherapy by the SWOG 9008/INT0116 scheme [47] is preferred in public health centres [48] and with a lower rate of the use of postoperative chemotherapy [43]. However, recent efforts like the PRECISO study (NCT01633203) seek to evaluate the clinical aspects of the introduction of perioperative chemotherapy in beneficiaries of the public system [49]. In private centres that carry out postoperative radiochemotherapy, there is access to new technologies such as intensity-modulated radiotherapy, through which a better distribution of dose in the target volume and lower dose of radiation in risk organs can be achieved, thus reducing the toxicity of the treatment [50]. The management of patients with metastatic disease is quite similar in the two systems, offering them palliative chemotherapy with schemes based on platinum salts combined with fluoropyrimidine [51, 52]. Despite the differences in the resource potentials when comparing the public sector and the private sector, the multidisciplinary work, the experience of this disease and the concentration of patients in hospitals with medical specialists have achieved good results in the survival expectancy rates and the quality of life of patients with gastric cancer in Chile.

## Conclusion

The present epidemiological changes both worldwide and in Chile have encouraged researchers to look for answers that enable us to understand the new observable phenomena regarding gastric cancer. As Chile is a highly endemic country for this disease, the study of the determining factors for gastric cancer in our population enables us to recognise findings present in other populations, like the relationship between infectious factors such as *H. pylori* and EBV, high salt consumption and racial factors such as Mapuche descent.

In reference to this last point, given the extensive multiethnic character of our population, and thanks to the new tools that provide genome sequencing and molecular techniques, these make the genetic and molecular model of the gastric cancer in the Chilean population an attractive topic to research. This is how translational medicine gains an important value, enabling the identification of epigenetic mechanisms such as the methylation of the hMLH1 and Reprimo genes, opening up an important line of research as it generates knowledge that could identify the presence of gastric cancer in early stages and predict the response to existing therapies. Given the lack of systematic screening in our country and the high rate of diagnosis in advanced stages of the disease, the basic science and clinical research that has been carried out in recent years in Chile has become even more relevant. Despite efforts made by the state in pursuit of improving diagnosis and appropriate treatment, there are still evident gaps to bridge, which not only need economic resources but also multidisciplinary efforts between the different parties dedicated to the research and treatment of this disease, in order to reach the ultimate goal, which is to improve the health of our population, provide better quality of life and meet the health demands of our patients.
